# Effect of drying procedures on the physicochemical properties and antioxidant activities of polysaccharides from *Crassostrea gigas*

**DOI:** 10.1371/journal.pone.0188536

**Published:** 2017-11-27

**Authors:** Sijie Hu, Guanhua Zhao, Yaxu Zheng, Min Qu, Qiao Jin, Changqing Tong, Wei Li

**Affiliations:** College of Food Science and Engineering, Dalian Ocean University, Dalian, P. R. China; Kermanshah University of Medical Sciences, ISLAMIC REPUBLIC OF IRAN

## Abstract

*Crassostrea gigas* polysaccharides (CGP) were obtained by different drying methods: freeze-drying (FD), spray-drying (SD) or rotary evaporation-drying (RED). The physicochemical properties of CGP were evaluated on the basis of polysaccharide content, protein content, color characteristics, FT-IR spectroscopy, differential scanning calorimetry (DSC), and scanning electron microscopy (SEM). Antioxidant activities were researched three different free radicals, including DPPH free radicals, ABTS free radicals and reducing power. The results demonstrated that FDCGP, SDCGP and REDCGP have different physicochemical properties and antioxidant activities. Contrasted with FDCGP and REDCGP, SDCGP exhibited stronger antioxidant abilities. Therefore, considering the polysaccharides appearances and antioxidant activities, the spray drying method is a decent selection for the preparation of such polysaccharides, and it should be selected for application in the food industry.

## Introduction

*Crassostrea gigas*, one of China’s major cultured shellfish, with production reaching 1083 thousand metric tons in 2015[1], is deemed a precious food and medicine resource[2]. *C*. *gigas* is rich in polysaccharides. Previous study has found that a *C*. *gigas* polysaccharide is a evenly glucose polymer, comprising primarily of →4)-α-D-Glc-(1→, with few →3,4)-β-D-Glc-(1→ and →2,4)-β-D-Glc-(1→ branched units[3]. The studies employed hot-water extraction, isoelectric precipitation, hydrolysis and ultrafiltration, without utilizing ethanol to extract *C*. *gigas* polysaccharides[4]. At present, studies of CGP primarily relate to functional activities, such as antimicrobial[5], antioxidant[6], anti-tumor[7], immune-stimulatory[8], and antihypertensive[9] functions. To date, most studies have only paid attention to extraction methods. During the manufacture of CGP, drying technology is also the last and most important process. In addition, drying methods have a important impact on the biological activities[10].

Several methods have been utilized to dehydrate the polysaccharides, including freeze-drying (FD), spray-drying (SD) and rotary evaporation-drying (RED). FD is normally combined with vacuum technology and used to dehydrate frozen polysaccharides. According to Minjares-Fuentes et al. (2017) and Zhao et al. (2015), drying methods assumed a significant part in the physicochemical properties and antioxidant activities. However, the drying product is easily fluffy and irregular in a vacuum freeze dryer. RED usually removes water by evaporation and leads to mass and energy transfer. However, following ebullition, the appearance of the samples would be changed and varied. SD has the characteristics of high temperature and short contact time, can produce polysaccharides with low water activity, it is ideal for drying polysaccharides[13]. Thus, it is essential to evaluate the effect of drying methods on the biological activities and physical appearances of polysaccharides obtained from *C*. *gigas*.

The studies regarding drying methods on CGP were finite. To the best of our knowledge, the impact of industrial-scale drying methods on the physical appearance and antioxidant activities of CGP have not been reported to date. Therefore, the aims of the current study are to evaluate the physical appearance and antioxidant activities of CGP treated by different drying methods (freeze, spray, and rotary evaporation drying) to identify the most proper drying method for the production of polysaccharide powder.

## Materials and methods

### Materials

*C*. *gigas* was purchased from a local Changxing market in Dalian, China. After physically expelling the internal organs and shells, the sample was washed preceding extraction.

2,2-Diphenyl-1-picryl-hydrazyl (DPPH), 2,2-azino-bis-(3-ethyl-benzthia-6-sulfonic acid) (ABTS) and 2,4,6-tri(2-pyridyl)-s-triazine (TPTZ) were purchased from Sigma Chemical Co. (St. Louis, MO, USA). All chemicals were of analytical grade or better.

### Polysaccharides extracted from *C*. *gigas*

*Crassostrea gigas* polysaccharides (CGP) were prepared by the method of Shi et al. (2015). The ultimately retentate solution was later separated into three batches for subsequent drying.

### Drying methods

Drying procedures of CGP retentate solution were conducted using three different methods, including freeze-drying (FD), spray-drying (SD) and rotary evaporation-drying (RED). The CGP retentate solution was lyophilized in an FD-1 vacuum freeze dryer (Botong Co., Shanghai, China) at −50°C for 72 h. The CGP retentate solution was sprayed in a spray dryer (Wode Co., Shanghai, China) at 180 and 95°C inlet and outlet temperatures. RED was done by a rotavapor (Yarong Co., Shanghai, China) at 55°C for 2 h, and it was moved to an electric heating air-blowing drier (Suhai Co., Yancheng, China) at 100°C for 12 h. The polysaccharides obtained by freeze-drying, spray-drying and rotary evaporation-drying were named as FDCGP, SDCGP and REDCGP, respectively.

### Polysaccharide and protein content

The total polysaccharide content was measured by phenol-sulfuric acid method [14] with glucose used as the standard. The reducing sugar content was detected using 3,5-binitro salicylic acid[15]. The protein content was estimated by Lowry’s method [16] using bovine serum albumin as a standard.

### Measurement of the molecular weight

The molecular weights of FDCGP, SDCGP or REDCGP were obtained by a gel permeation chromatography system with a TSK-gel G4000PW_XL_ column (7.8 mm×30 cm) in H_2_O. The dextrans with molecular weights of 10, 70, 80 and 500 kDa were used as standard water-soluble polysaccharides.

### Color characteristics

Color determination was performed on the surface of FDCGP, SDCGP and REDCGP. A handheld CR-400 chroma meter (KONICA MINOLTA Co., Tokyo, Japan) was used with an illuminant of D65. Using the CIE L* (lightness), a* (deviation towards red or green), b* (deviation towards yellow or blue) coordinates, the psychophysical magnitudes of h _ab_ * (hue) and C _ab_ * (chroma) were calculated using equations (Eqs) (1) and (2), respectively.

$$C_{\mathrm{ab}}*=\sqrt{{a*}^{2}+{b*}^{2}}$$ (1)

$$h_{ab}*=arctg(b*/a*)$$ (2)

The overall color differences (ΔE) were calculated using Eq (3).

$$\Delta E=\sqrt{{\left(\Delta a*\right)}^{2}+{\left(\Delta b*\right)}^{2}+{\left(\Delta L*\right)}^{2}}$$ (3)

### Scanning electron microscopy

The morphological characteristics of the samples were recorded using an S-4800 scanning electron microscope (Hitachi, Tokyo, Japan). Samples were inspected on a Q150TES (Quorum, United Kingdom) at a tension ranging from 5 to 15 kV.

### FT-IR spectroscopy

FT-IR spectroscopy measurement was used a 600-IR instrument (Agilent Technologies Co., USA) in the range of 4000 to 400 cm^-1^.

### UV spectroscopy

The polysaccharides were made into 1 mg/mL aqueous solutions. UV spectra were generated using a Lambda 25 UV spectrophotometer (Perkin-Elmer, USA) in the range of 200–700 nm.

### Thermal analysis

The thermal behavior of the polysaccharides was tested using a differential scanning calorimetry (DSC) (Q20, TA, USA). The samples were placed in the sample pan and executed over a temperature range of 20 to 400°C at 20°C/min under a nitrogen atmosphere.

### Assay for antioxidant activity

#### Assay for DPPH radical scavenging activity

The DPPH radical scavenging assay was conducted on the basis of a literature procedure with a few modifications [17]. DPPH (2 mL, 0.1 mM) solution was added into 2 mL of a series of concentrations of the sample solution. The reaction mixture was shaken well and incubated for 30 min, and the absorbance was measured at 517 nm.

#### Assay for ABTS radical scavenging activity

The radical scavenging activity of sample was evaluated using the method previously carried out by Wang, Chang, Inbaraj, & Chen, (2010)[18]. The ABTS radical (ABTS^+^) was generated by mixing an ABTS (7.4 mM) solution with a potassium persulfate (2.6 mM) aqueous solution and then leaving the mixtures in the darkness for 16 h. A total of 1.6 mL freshly prepared ABTS^+^ solution was added to 0.4 mL of the sample, and the mixture was shaken and allowed to stand at room temperature in the dark for 6 min. The absorbance was measured at 734 nm.

#### Assay of reducing power

The reducing power of the sample was carried out according to the procedure of Oyaizu, (1986) with slight modifications[19]. Various concentrations of the sample solution, 2.5 mL phosphate buffer (0.2 M, pH 6.6) and 1.0 mL potassium ferricyanide (1%, w/v) were mixed and incubated at 50°C for 20 min. Next, 5.0 mL trichloroacetic acid (10%, w/v) and 0.5 mL fresh ferric trichloride (0.1%, w/v) were added to the reaction mixture. The total mixture was incubated for 10 min, and the absorbance was measured at 700 nm.

### Statistical analysis

All data were performed at least in duplicate, and analyses of all samples were performed in triplicate and averaged. Statistical analysis involved using SPSS software version 11.0 (Chicago, USA). The results were presented with three replicates ± SD (standard deviation).

## Results and discussion

### Physicochemical properties

**Fig 1:**
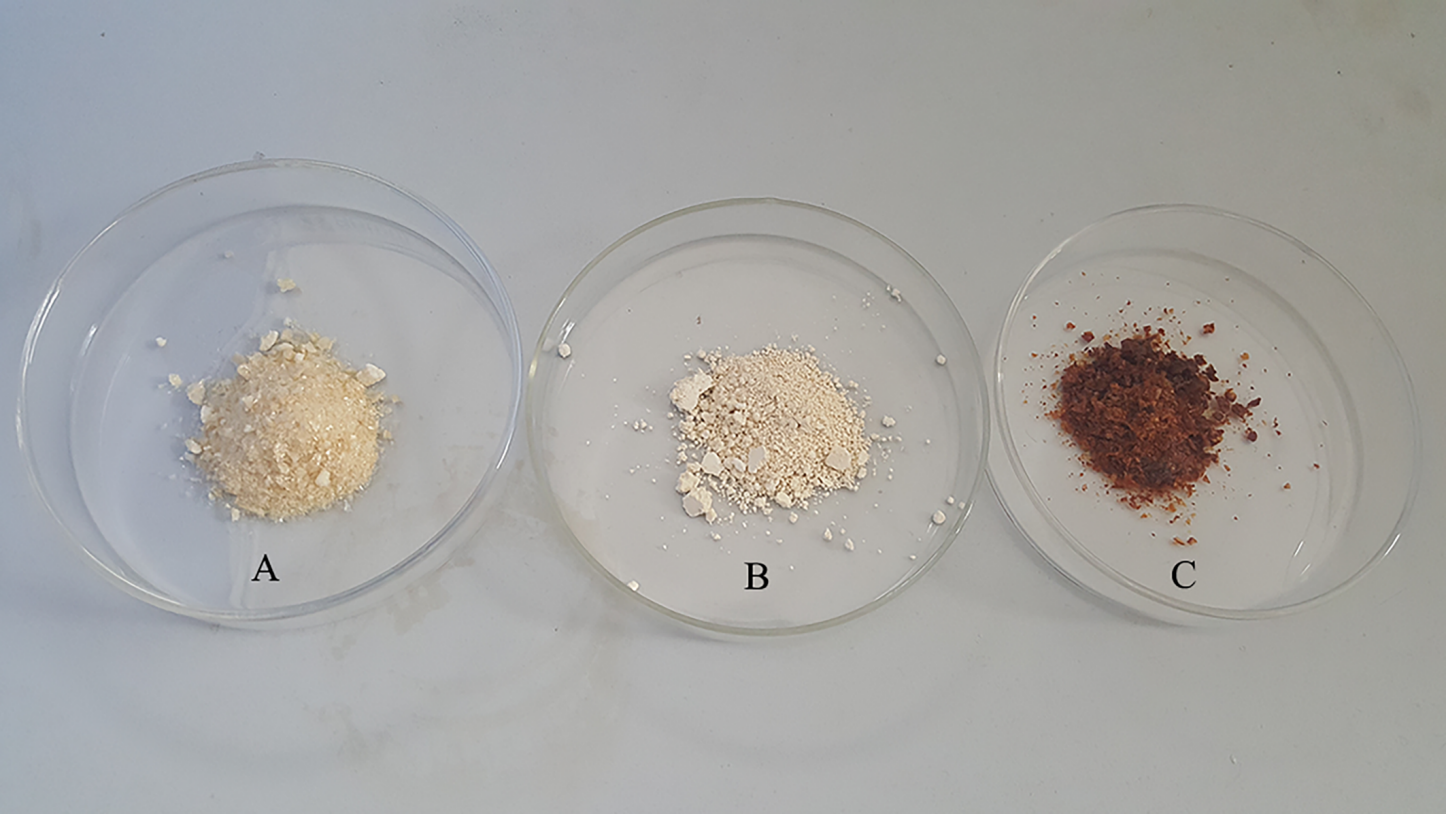
Photographs the polysaccharides from *C*. *gigas* (A: FDCGP; B: SDCGP; C: REDCGP).

All photographs of the samples are shown in Fig 1. The appearance of FDCGP, SDCGP and REDCGP evidently differed. The physical characteristics of CGP got by three methods are summarized in Table 1. All samples could be dissolved in H_2_O, and they were insoluble in organic solvents. The bioactivity of the polysaccharides mainly from chemical composition, molecular weight, types of glycosidic bond and structure to understand[20]. The polysaccharides contents in FDCGP, SDCGP and REDCGP were 47.7±1.8%, 41.8±1.0% and 37.0±0.9%, respectively. All FDCGP, SDCGP and REDCGP included the reducing sugar. The contents of the reducing sugar were listed in the order of SDCGP˃ FDCGP˃ REDCGP, and were 3.1±0.2%, 1.9±0.1% and 1.5±0.2%, respectively. This might be attributed to loss of the contents by different methods [21,22]. Therefore, the polysaccharide’s content was certain impacted by the various drying methods. In addition, the protein content of the samples was approximately 13.5%. This result was consistent with previous reports[3,23].

**Table 1:** Physicochemical properties of polysaccharides by different methods.

	Polysaccharide content (%)	Protein content (%)	The reducing sugar (%)	MW distribution
FDCGP	47.7±1.8	13.5±0.5	1.9±0.1	1.2×10^7^,1.5×10^5^
SDCGP	41.8±1.0	13.3±0.4	3.1±0.2	1.2×10^7^,7.3×10^4^
REDCGP	37.0±0.9	13.9±0.4	1.5±0.2	1.2×10^7^,7.3×10^4^

The molecular weights in FDCGP, SDCGP and REDCGP were in the following order: FDCGP˃ SDCGP and REDCGP. The molecular weight of FDCGP was higher than that of SDCGP and REDCGP, revealing that freeze-drying method was easily aggregated the polysaccharide molecules. The results may be due to the differing drying conditions, thermal degradation by high temperature and the shear-forces present in the spray-drying chamber may promoted a significant reduction of the molecular weight of polysaccharides. It is generally believed that high temperature may destroy the polysaccharide aggregation and shear-forces may cause chain scission, result in a molecular weight reduction of branched molecules[24]. Previously, Minjares-Fuentes et al. has reported that the molecular weight of polysaccharide acemannan from spray-drying samples was decreased significantly[11]. Medina-Torres et al. found that the reduction of the molecular weight of polysaccharides from Aloe vera mucilage is largely associated with high temperature of drying process[25]. And research determined that the polysaccharides with small molecular weights frequently shown more abundant biological activities than those with large ones [26].

### Physical appearance

#### Color characteristics

The values analyzed in the color determinations (L*, a*, b*, h_ab_*, C_ab_*, ΔE*) are summarized in Table 2. It was observed that SDCGP was brighter than FDCGP and REDCGP. Similar results has been reported in other studies[11,27]. The overall color differences (ΔE*) were calculated in order to quantify the potential color change of the samples by various drying methods. The results show that SDCGP exhibited a minor color change. A low ΔE* value is often indicative of an ideal color. The REDCGP had the lowest L value (indicating darkest color), which can be due to Maillard reaction and caramelization of sugars caused in CGP[27,28]. On the other hand, SDCGP appeared to have the highest L value. While the temperature during spray drying process was very high, but the spray time is very short and therefore the color remained bright[29]. Similar results have also been observed in mango powder and Aloe vera samples[11,27]. The highest lightness and minimal color change of polysaccharides produced by spray-drying suggests that SD is suitable for producing high quality CGP.

**Table 2:** Changes in color parameters L, a*, b*, C_ab_*, h_ab_* and ΔE* in the different drying procedures.

	L	a*	b*	C_ab_*	h_ab_*	ΔE*
FDCGP	71.1±0.1	1.8±0.0	1.4±0.0	2.3±0.0	0.7±0.0	23.8±0.1
SDCGP	82.6±0.2	1.5±0.0	1.2±0.2	1.9±0.1	0.7±0.1	12.4±0.2
REDCGP	57.8±0.0	2.0±0.1	-1.2±0.1	2.4±0.1	-0.6±0.1	37.2±0.0

### Scanning electron microscopy

**Fig 2:**
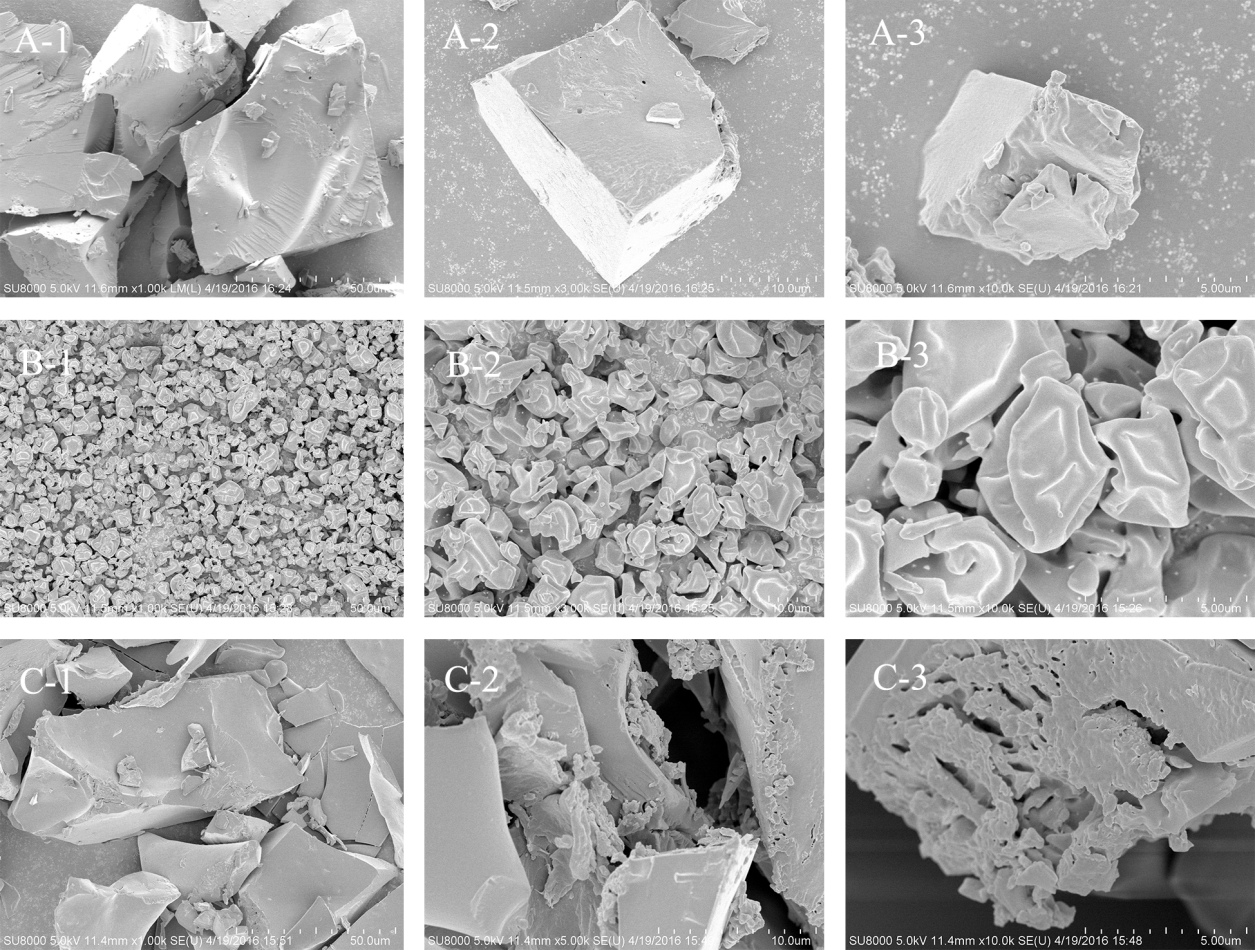
SEM of polysaccharides by different methods. (A) FDCGP: A1×50; A2×10; A3×5. (B) SDCGP: B1×50; B2×10; B3×5. (C) REDCGP: C1×50; C2×10; C3×5.

The morphology of the samples from CGP are summarized in Fig 2. Different drying methods may affect the surface topography and structure of polysaccharides [30]. The morphological differences among the samples were observed. SDCGP is smaller and more uniform in size than FDCGP or REDCGP and presented oval shape and smooth surface particles (Fig 2B). The morphology of particles was smooth indicating more bioactive and antioxidative activity because of lesser surface area[31,32]. And Ma et al. and Choi et al. had reported that smaller particles could lead to the changes in molecular weight, intermolecular distance and interconnection[33,34]. Therefore, it could explain the smaller molecular weight of SDCGP compared with the others. The morphology reported for samples by spray-drying has also been described in the literature[11,35–37]. In the case of FDCGP, it has a smooth and angular edge. Caparino et al. and Minjares-Fuentes et al. described a similar morphology in other samples by freeze-drying[11,27]. However, REDCGP is loose and irregular. These observations are in agreement with the microstructure of different drying methods as described[38–40]. It was very important that the wettability and solubility of sample were affected the structure of sample particles in powder instant production development.

### FT-IR spectroscopy and UV spectroscopy

**Fig 3:**
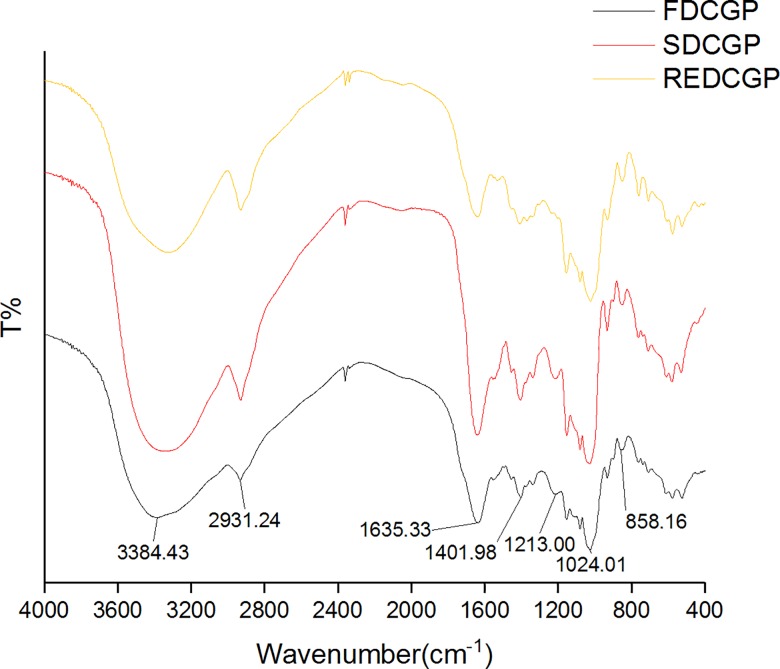
FT-IR spectra of polysaccharides by different methods.

There was no visible difference between the FT-IR spectra of the three samples by different drying methods (Fig 3). The result shows that FDCGP, SDCGP and REDCGP had peaks that were typical of a polysaccharide, and this result was corresponded with the findings of previous studies [4]. The broad band at 3384.43 cm^−1^ was attributed to the stretching of hydroxyl groups. The peak at 2931.24 cm^−1^ could be assigned to C−H stretching of the alkane, and the band at 1401.98 cm^−1^ was due to C−H bending vibration. The peak at 1635.33 cm^−1^ is attributed to the bound water. The peak appearing at 1213.00 cm^−1^ was due to O−O stretching or a protein structure [41,42]. The absorption peak at 1024.01 cm^−1^ showed that the samples possessed the pyranose ring skeleton. The characteristic peak at 858.16 cm^−1^ indicates the presence of the α-configuration of D-glucan [4,42]. The results showed that the polysaccharide structure was not destroyed by three drying methods. The UV spectra of the three polysaccharides were similar, but no identified new double and aldehyde groups (S1 Fig).

### Thermal analysis

**Fig 4:**
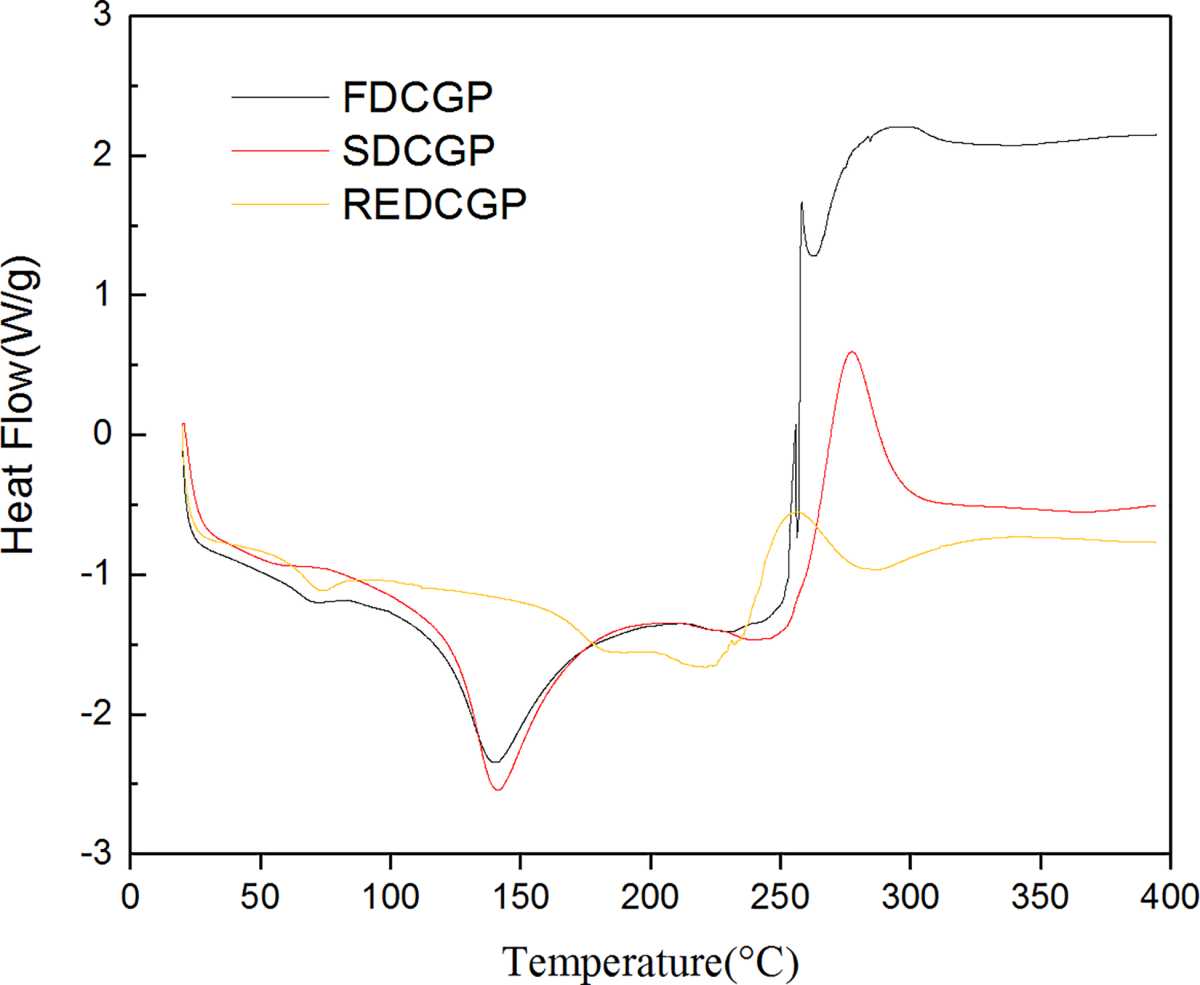
DSC curves of polysaccharides by different methods.

Differential scanning calorimetry (DSC) was used to measure the occurrence of exothermal or endothermal changes with increased temperature[6] and it was fulfilled so as to apprehend the thermal behavior of the samples[43]. As shown in Fig 4, the DSC curves of FDCGP and SDCGP have similar shapes. The DSC thermogram displayed an initial endothermic phase and an exothermic phase. The first event of FDCGP and SDCGP, resulting in an endothermic peak, was revealed at a range of 130 to 140°C. This event is related to the presence of the dehydration and impurities in the samples, which occurs over a range of temperatures. However, REDCGP showed no variation at the same temperature, which may relate to the drying method used. A second event, corresponding to an exothermic transition, was observed at 250–300°C, which is led to the thermal decomposition of the samples[44]. It can also be seen from DSC that SDCGP is thermally more stable, which may be caused by structure of the polysaccharides, molecular weight, degree of polymerization and branching of the samples[45].

### Antioxidant activities

#### Scavenging activity on DPPH radicals

**Fig 5:**
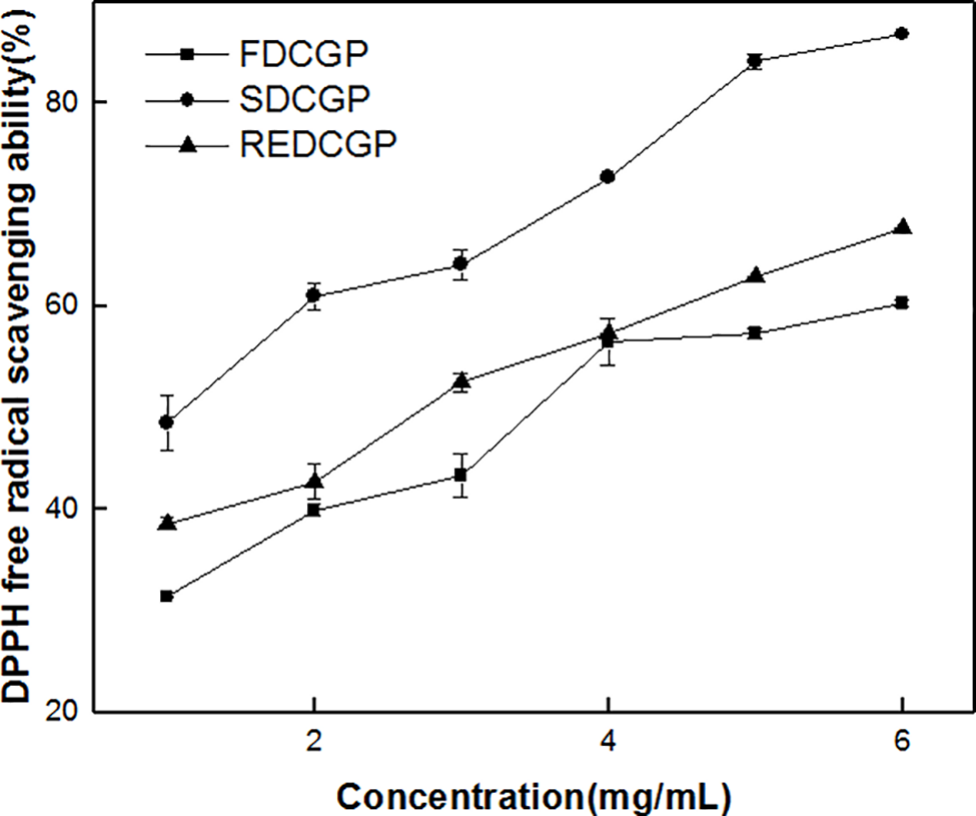
Scavenging effects of polysaccharides by different methods on the DPPH radical. The results were presented as the mean ± SD (n = 3).

The DPPH radical is widely would measure the free radical scavenging activities of antioxidants[46], and the DPPH solution purple in the face of proton free radical scavengers rapidly disappeared[17]. Fig 5 illustrates the scavenging rate of all samples showing scavenging ability on DPPH radicals. For FDCGP, SDCGP and REDCGP, the DPPH radical scavenging abilities depended on concentration. According to the report by Ma et al. and Chen et al, the DPPH radical ability of polysaccharide might be related to monosaccharide component, molecular weight, and conformation[21,47]. Furthermore, the low molecular weight polysaccharides from *P*. *cruentum* was reported to be effective in the oxide, but the high molecular weight polysaccharides had no obvious antioxidant activities[48]. Among the three polysaccharides, SDCGP exhibited higher DPPH radical scavenging rate than FDCGP and REDCGP at every concentration point probably because of higher molecular weight of SDCGP. The result suggested that the DPPH radical scavenging capabilities were affected by drying methods.

#### Scavenging activity on ABTS radicals

**Fig 6:**
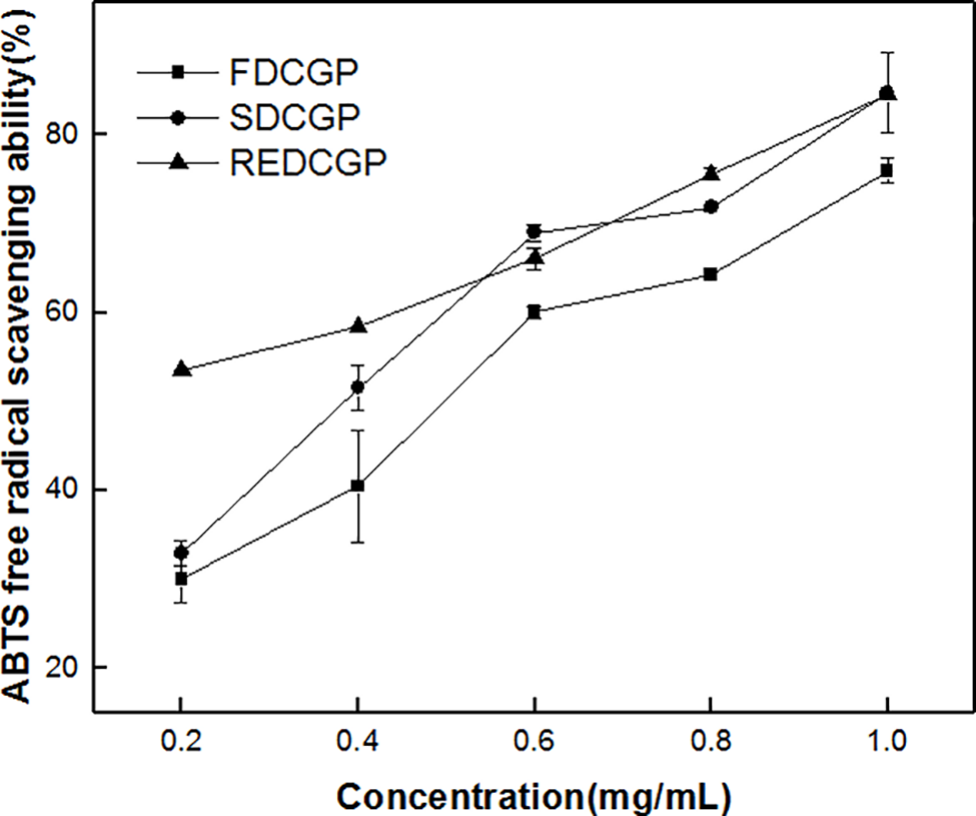
Scavenging effects of polysaccharides by different methods on ABTS radical. The results were presented as the mean ± SD (n = 3).

ABTS^+^ is a free and stable radical cation, which could be accepted a hydrogen atom or an electron as an antioxidant. It has been used to study the antioxidants[49]. The scavenging ability on ABTS free radical is shown in Fig 6. All samples were greatly scavenged the ABTS radicals in a dose-dependent manner. However, the ABTS scavenging ability decreased in the following order: FDCGP˃REDCGP˃SDCGP. The result indicated that SDCGP shown the highest scavenging power on ABTS radical in contrary to the weakest one of FDCGP. The scavenging the ABTS radicals was well correlated with hydroxyl group in polysaccharides. Not only could hydroxyl groups of the polysaccharides be easily oxidized under the aerobic condition, but also the ABTS radicals scavenging capacities of the polysaccharides dried in oxygen environment were obviously decreased due to the oxidation of the hydroxyl groups[26]. In addition, the reaction of ABTS radicals with the active hydroxyl groups could be inhibited by the hydrogen bonds formed between intermolecules or intramolecules of the polysaccharides [50]. Compared with freeze-drying or rotary evaporation-drying process, CGP was dehydrated by spray-drying under the aerobic condition. This result was in accord with present study, but the mechanisms require further investigation.

#### Reducing power

**Fig 7:**
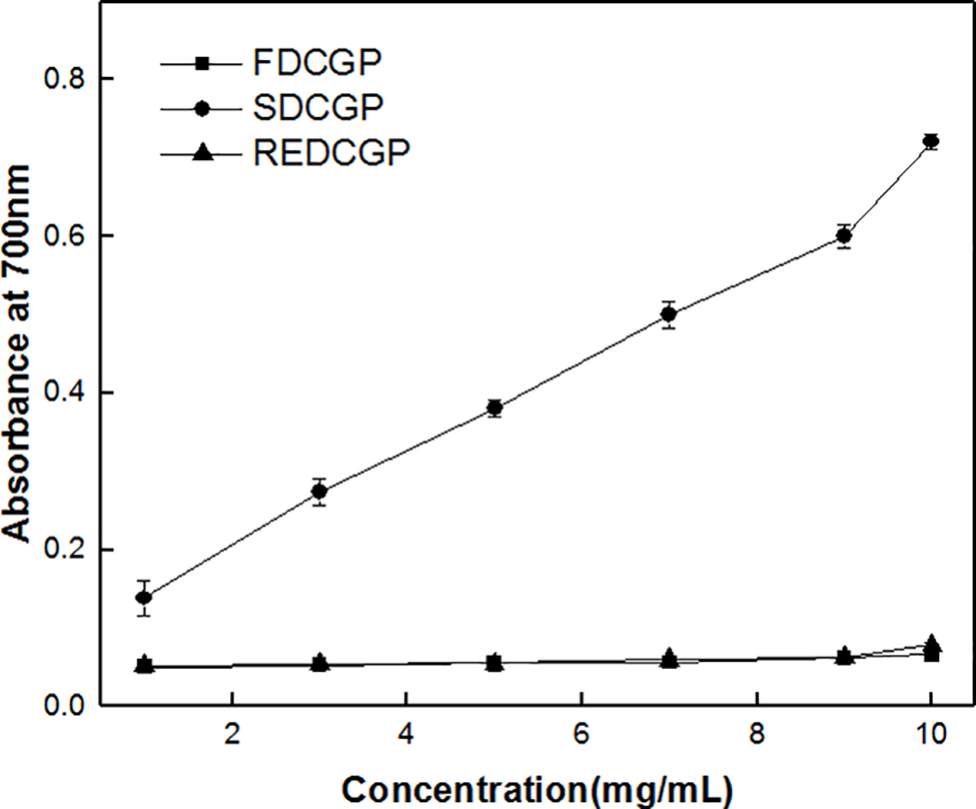
Reducing power of polysaccharides by different methods. Data were presented as the mean ± SD (n = 3).

The reducing power works as an important potential index of antioxidants[51]. When reductones are present, it is possible to exert antioxidant actions by breaking the free radical chains by donating hydrogen atoms[52,53]. Fig 7 describes the reducing power of all polysaccharides. Compared with SDCGP at every concentration point, FDCGP and REDCGP exhibited lower reducing powers. The result showed that spray-drying could maximally retain the polysaccharides’ antioxidant activities. The research found that the presence of reductones in the polysaccharides played an important role in the reducing properties[54]. Moreover, spray-drying had good effect on preserving antioxidant activity because it can dehydrate the sample very apace while producing the smoother particle size distribution, which meant little antioxidant activity damage during the drying process[12].

## Conclusions

In this study, three drying procedures significantly impact the physicochemical properties and antioxidant activities of the polysaccharides. The effect on the physicochemical characteristics and antioxidant activities of polysaccharides obtained from *C*. *gigas* by various drying methods has rarely been published. Among three polysaccharides, spray-drying had the best physical appearance, a more uniform morphology and the strongest scavenging effects, which was a good choice for producing of polysaccharides. Overall, spray-drying was the most appropriate method for producing high quality *C*. *gigas* polysaccharides.

## Supporting information

S1 Fig UV spectra of polysaccharides by different methods. (TIF)

S1 File The raw data. (ZIP)
