# Supplementary figures and images for: Effect of drying procedures on the physicochemical properties and antioxidant activities of polysaccharides from Crassostrea gigas
Source: PLoS One. 2017 Nov 27;12(11):e0188536. doi: 10.1371/journal.pone.0188536 (PMC5703540; doi:10.1371/journal.pone.0188536)

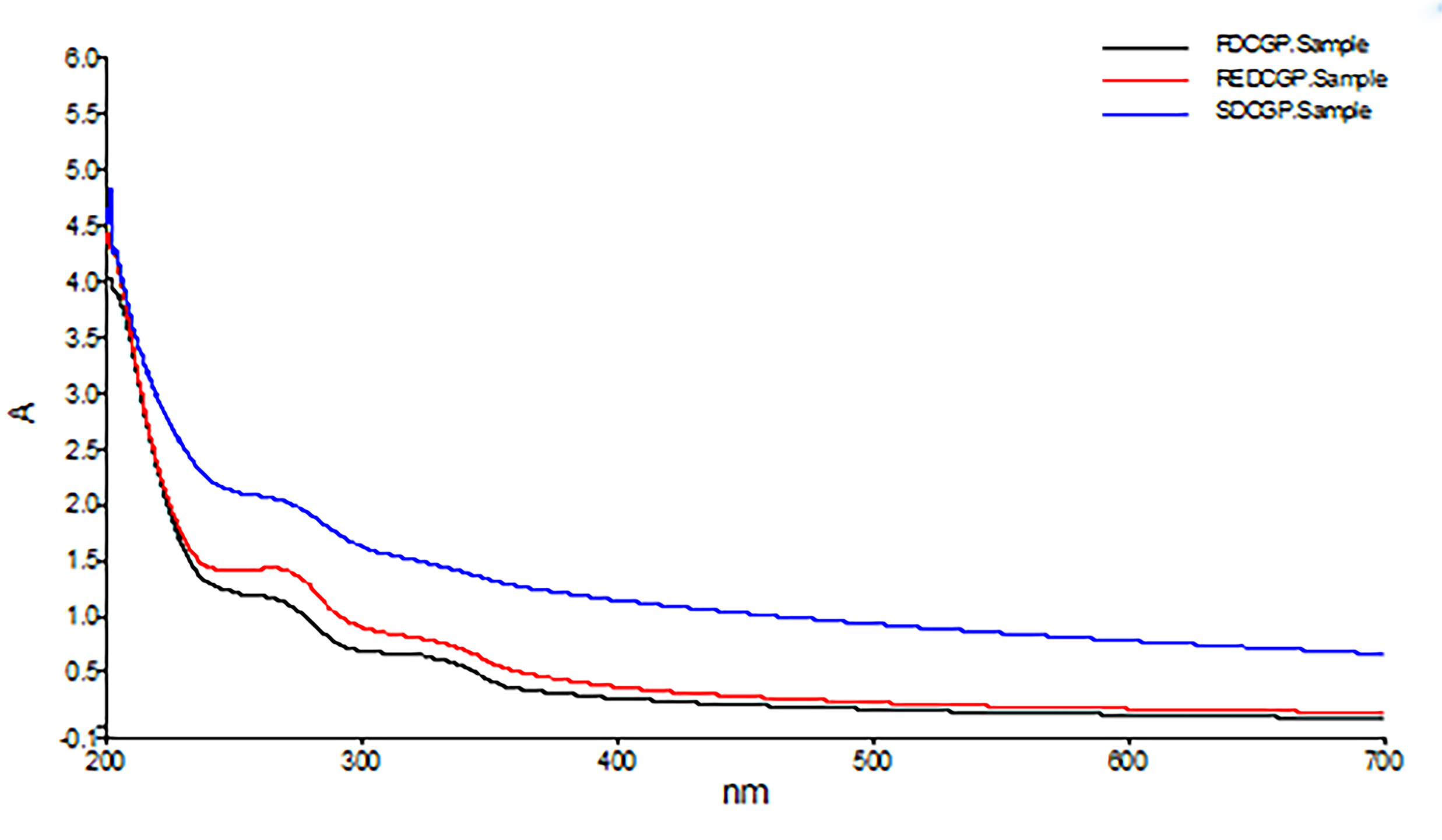

Supplement: S1 Fig — (TIF) [file pone.0188536.s001.tif]

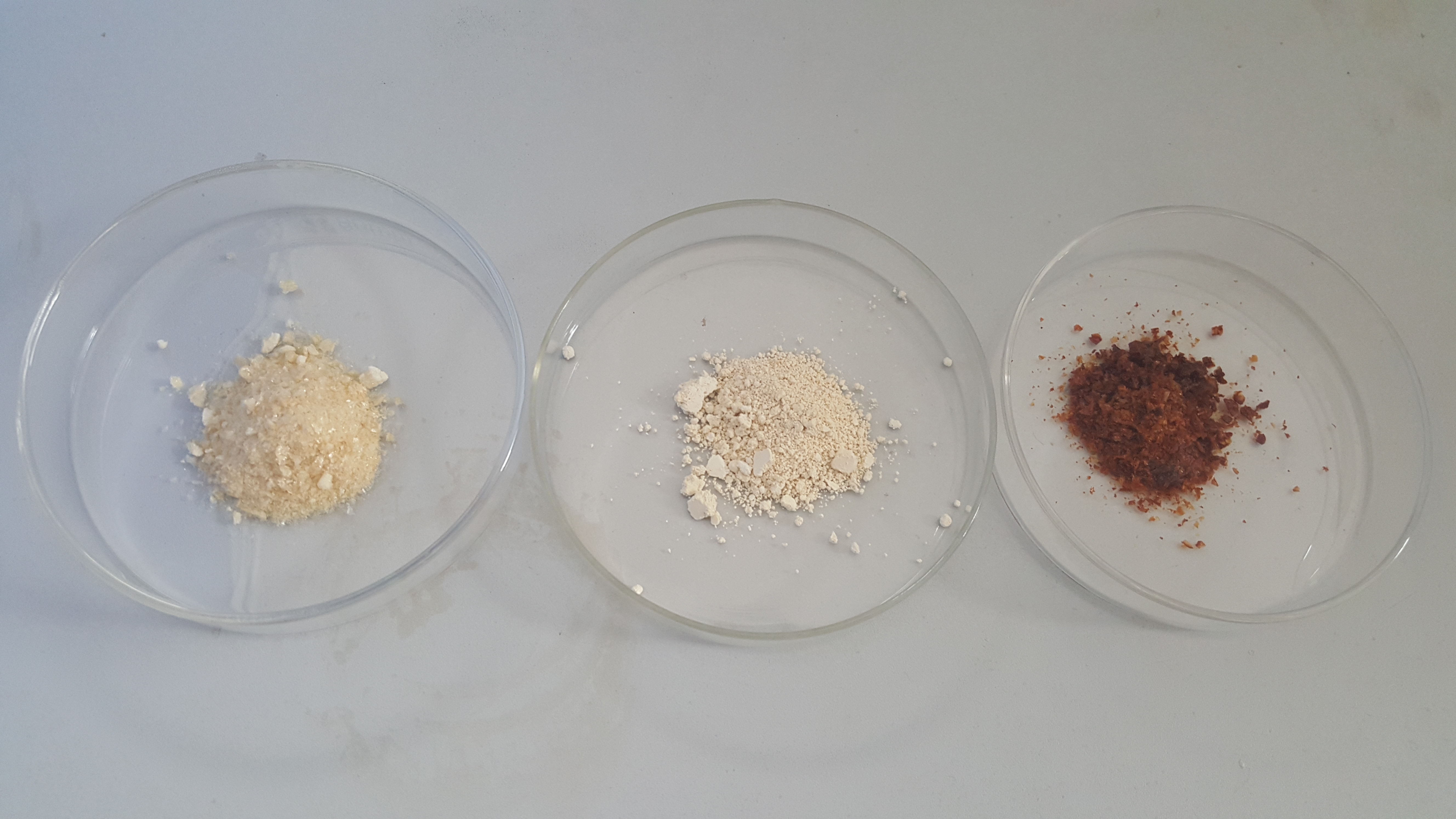

Supplement: S1 File — (ZIP) [file pone.0188536.s002.zip › data/20170628_143358.jpg]

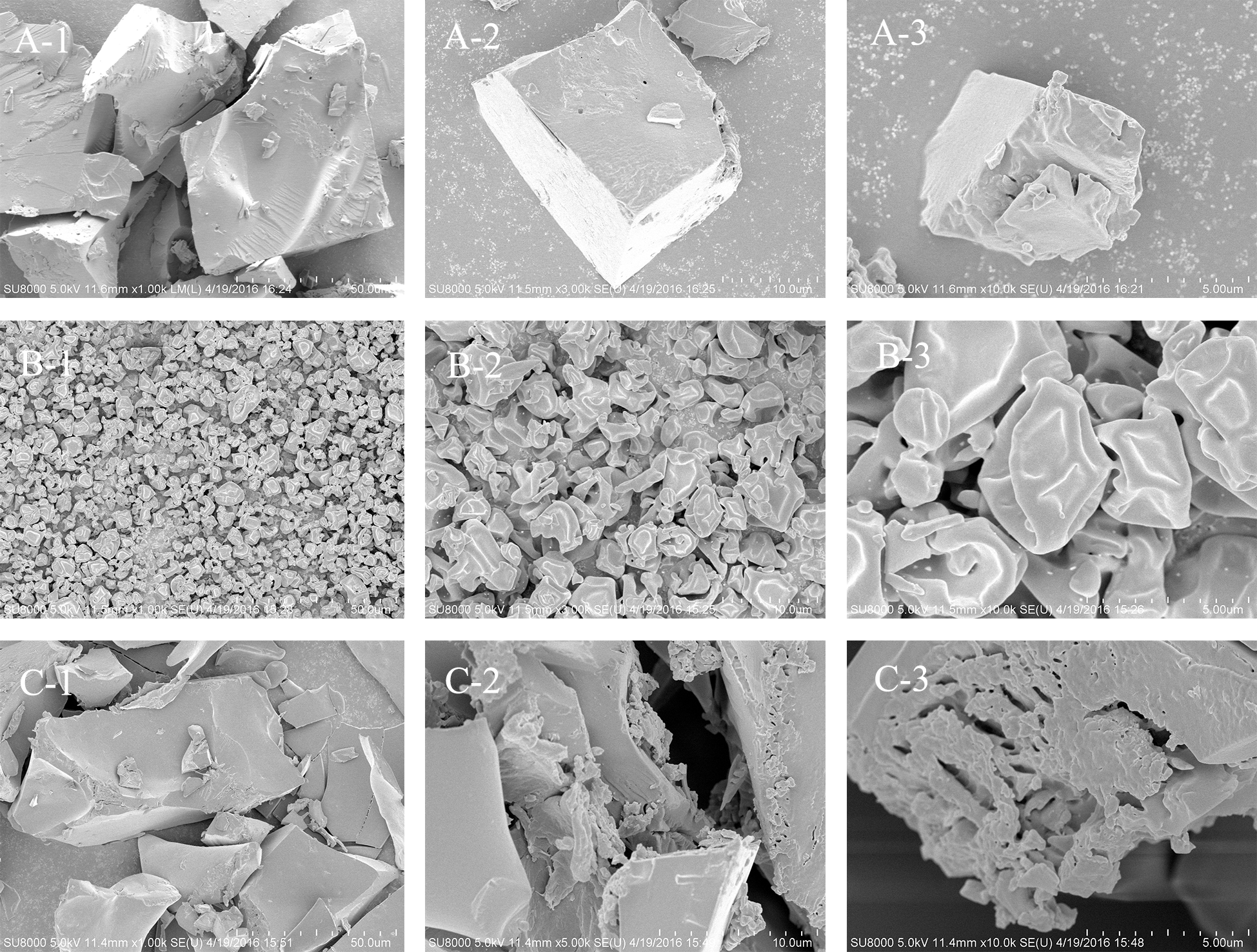

Supplement: S1 File — (ZIP) [file pone.0188536.s002.zip › data/SEM╕▒▒╛.tif]

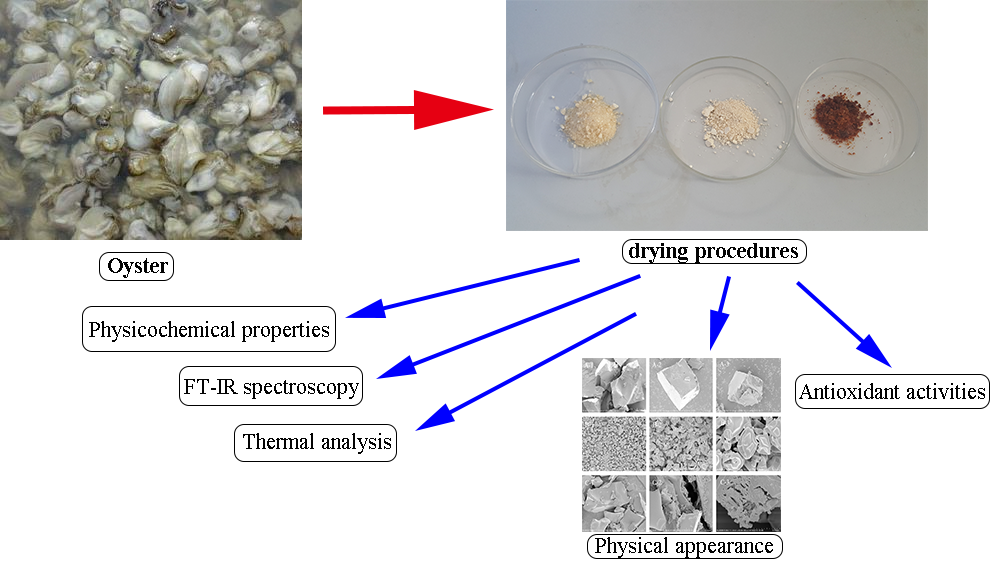

Supplement: S1 File — (ZIP) [file pone.0188536.s002.zip › data/TOC Graphic.tif]

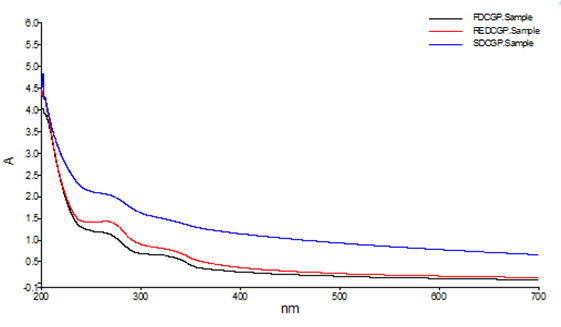

Supplement: S1 File — (ZIP) [file pone.0188536.s002.zip › data/UV/UV-2.tif]

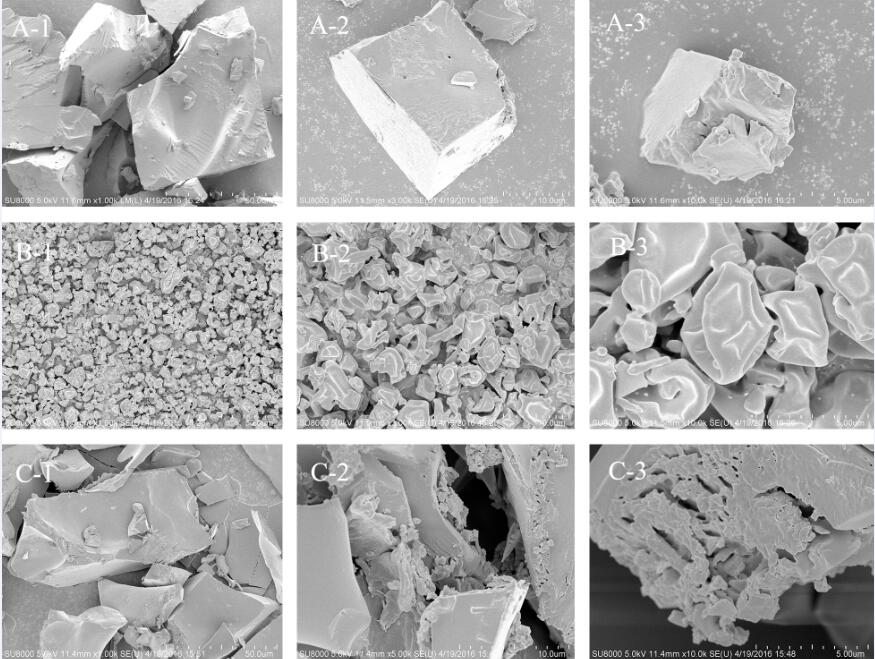

Supplement: S1 File — (ZIP) [file pone.0188536.s002.zip › data/sem1.jpg]

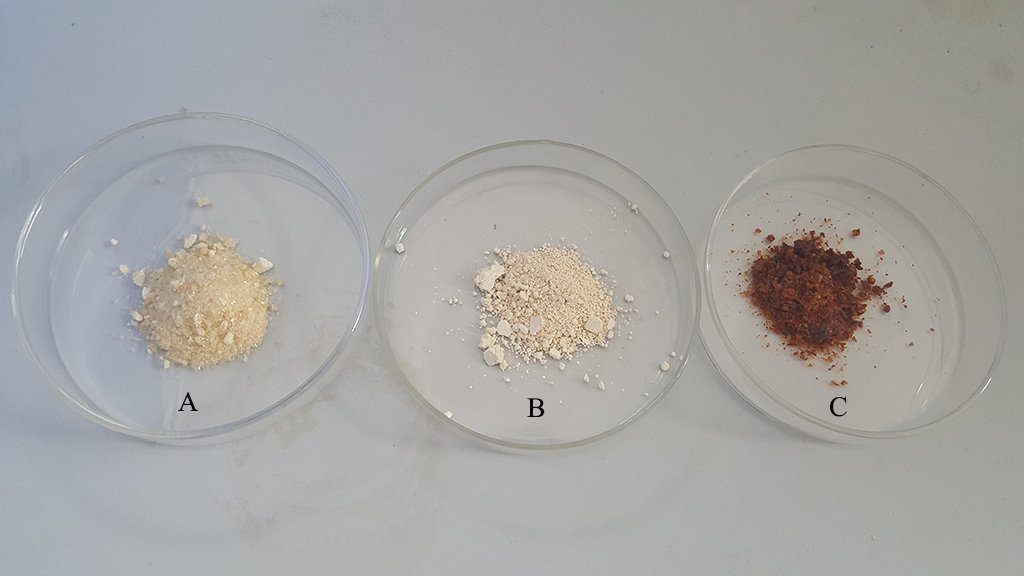

Supplement: S1 File — (ZIP) [file pone.0188536.s002.zip › data/═Γ╣█.tif]
